# BRD4 as a Therapeutic Target in Pulmonary Diseases

**DOI:** 10.3390/ijms241713231

**Published:** 2023-08-25

**Authors:** Xia Guo, Ayobami Olajuyin, Torry A. Tucker, Steven Idell, Guoqing Qian

**Affiliations:** Department of Cellular and Molecular Biology, The University of Texas Health Science Center at Tyler, Tyler, TX 75708, USA; xia.guo@uthct.edu (X.G.); amolajuy@utmb.edu (A.O.); torry.tucker@uthct.edu (T.A.T.); steven.idell@uthct.edu (S.I.)

**Keywords:** BET, BRD4, inflammation, lung

## Abstract

Bromodomain and extra-terminal domain (BET) proteins are epigenetic modulators that regulate gene transcription through interacting with acetylated lysine residues of histone proteins. BET proteins have multiple roles in regulating key cellular functions such as cell proliferation, differentiation, inflammation, oxidative and redox balance, and immune responses. As a result, BET proteins have been found to be actively involved in a broad range of human lung diseases including acute lung inflammation, asthma, pulmonary arterial hypertension, pulmonary fibrosis, and chronic obstructive pulmonary disease (COPD). Due to the identification of specific small molecular inhibitors of BET proteins, targeting BET in these lung diseases has become an area of increasing interest. Emerging evidence has demonstrated the beneficial effects of BET inhibitors in preclinical models of various human lung diseases. This is, in general, largely related to the ability of BET proteins to bind to promoters of genes that are critical for inflammation, differentiation, and beyond. By modulating these critical genes, BET proteins are integrated into the pathogenesis of disease progression. The intrinsic histone acetyltransferase activity of bromodomain-containing protein 4 (BRD4) is of particular interest, seems to act independently of its bromodomain binding activity, and has implication in some contexts. In this review, we provide a brief overview of the research on BET proteins with a focus on BRD4 in several major human lung diseases, the underlying molecular mechanisms, as well as findings of targeting BET proteins using pharmaceutical inhibitors in different lung diseases preclinically.

## 1. Introduction

The bromodomain and extra-terminal domain (BET) family are known as epigenetic readers to regulate gene transcription. It has four members in mammals, i.e., bromodomain-containing protein 2, 3, 4 (BRD2, BRD3, and BRD4) and testis-specific BRDT. BET proteins have several conserved domains. All BET members share two tandem N-terminal bromodomains (BDs) and one C-terminal extra-terminal (ET) domain. The first and second BDs (BD1 and BD2) bind to acetylated lysine residues of nuclear proteins, through which they enhance activity of the transcriptional machinery and thus gene transcription. Compared to BRD2 and BRD3, BRD4 and BRDT also have a C-terminal domain (CTD). The CTD recruits the positive transcription elongation factor b (pTEFb), a cyclin-dependent kinase controlling elongation by RNA Polymerase II [1,2] to promote gene transcription. The ET domain may serve as another important transcriptional regulator through interaction with several cellular proteins including glioma tumor suppressor candidate region gene 1 (GLTSCR1), Jumonji domain-containing 6 (JMJD6), and nuclear receptor binding SET domain protein 3 (NSD3) [3,4,5]. Among these BET family members, BRD4 is the most studied. BRD4 and BRD2 knockout in mice are embryonically lethal [6,7]. BET regulates gene transcription through binding to acetylated lysine residues of nuclear proteins (e.g., histones, transcription factors, enhancers, super-enhancers, and others) to facilitate RNA polymerase II-dependent transcription [8]. Two important transcription factors, NF-κB and AP1, are known to be targets of BRD4 and important for its function [9,10]. BRD4 interactome analysis has uncovered approximately 100 proteins that are enriched in the BRD4 complex and responsive to respiratory syncytial virus (RSV)-infection and BRD4 inhibition [11], which is related to the BRD4’s acetyl-lysine binding bromodomains. In addition, several studies also reported the role of BRD4 in regulating alternative splicing to control cell differentiation, heat shock response, and innate immune response [12,13,14].

Advances in the development of BD-specific inhibitors [15] have shed novel light on the differential requirement of BD1 and BD2 for the maintenance and induction of gene expression in malignancy and inflammatory disease, which may direct future BET-targeting therapies. The testing of BET inhibitors (BETi) in different cancer types remains a research hotspot [15,16,17,18,19,20], which is important but will not be discussed here. Emerging evidence also suggests a critical role of BRD4 in the pathogenesis of several major lung diseases including acute lung injury such as acute respiratory distress syndrome (ARDS), asthma, pulmonary arterial hypertension (PAH), pulmonary fibrosis, and chronic obstructive pulmonary disease (COPD). In this review, we will focus on findings implicating BET proteins, particularly BRD4, in the pathogenesis of these pulmonary diseases, the implications of BRD4 in disease progression, and the underlying mechanisms they control.

## 2. Implications of BET Proteins in Pulmonary Diseases

Increasing evidence suggests the participation of BET proteins, particularly BRD4, in the development of pulmonary diseases (Table 1). The following sections will discuss the novel functions of BRD4 in pulmonary diseases based on findings from in vitro assays and preclinical models.

### 2.1. Acute Lung Inflammation

BET proteins are important for regulating inflammatory and immune responses. Of the first two BET inhibitors discovered, JQ1 and I-BET [60,61], I-BET was initially found to regulate macrophage-driven inflammation [61]. Several other BET inhibitors also demonstrate anti-inflammatory effects in the lung caused by lipopolysaccharide (LPS). For example, Chen and colleagues reported that the BETi CPI-203 remarkably suppressed Th17 cytokine production (IL-17A, IL-22) by T cells from the lungs of cystic fibrosis patients [21]. The authors further showed that CPI-203 treatment inhibited Th17 chemokines and cytokines in human bronchial epithelial cells derived from cystic fibrosis and control donors. In addition, CPI-203 decreased the inflammatory response in mice with acute *Pseudomonas aeruginosa* infection. During acute inflammation, adhesion of leukocytes to activated endothelial cells is an early event. A previous study showed that the BETi JQ1 attenuated the production of adhesion molecules and pro-inflammatory cytokines (e.g., IL-6 and IL-8) from human umbilical vein endothelial cells (HUVECs), and leukocyte adhesion to activated endothelial cells (ECs) induced by TNF-α in vitro [22]. The attenuation of NF-κB and p38 MAPK pathway activation by JQ1 may account for the effects of BET inhibition. Consistently, in LPS-induced acute lung inflammation, JQ1 pretreatment decreased leukocyte infiltration into the lung and suppressed the expression of VCAM-1, ICAM-1, and myeloid-related protein 14 (MRP14) [22]. In another study, the BD2-selective BETi RVX-297 also reduced proinflammatory mediators (IL-6 and IL-17) in the spleen and serum in a mouse model of LPS-induced acute inflammation [23]. The work by Liu et al. [24] showed that in polyinosinic:polycytidylic acid (poly(I:C))-induced acute lung inflammation mouse model, two selective BRD4 inhibitors (ZL0420 and ZL0454) more effectively blocked neutrophil infiltration into the lungs and cytokine expression in the lungs than that of JQ1 or RVX-208. In addition, ZL0420 and ZL0454 also demonstrated strong potency in inhibiting toll-like receptor 3 (TLR3)-dependent innate immune gene expression in human small airway epithelial cells (hSAECs). These several lines of evidence support BET inhibition as an attractive strategy in suppressing acute lung inflammation.

Respiratory viruses are key contributors to acute lung inflammation. Severe acute respiratory syndrome coronavirus 2 (SARS-CoV-2) is a strain of coronavirus that causes coronavirus disease 2019 (COVID-19) [62]. To search for potential drug targets, Gordon and colleagues identified 332 high-confidence protein–protein interactions between SARS-CoV-2 and human proteins. Interestingly, BRD2 and BRD4, but not other BET family members, were found on the list to interact with SARS-CoV-2 [25]. Further mechanistic studies by Vann et al. [63] showed that SARS-CoV-2 E protein interacts with human BRD4 through two different mechanisms, i.e., BD1 and BD2 of BRD4 binds to acetylated E protein at K53 and K63 sites and the ET domain of BRD4 interacts with an unacetylated motif (SFYVYSRVKNLN) of the E protein. Further, JQ1 and OTX015 treatment reduced SARS-CoV-2 infection in vitro. The data suggest that BRD4 contributes to SARS-CoV-2 infection. In addition, BRD2 and BRD3 regulate the expression of the viral entry receptor angiotensin-converting enzyme 2 (ACE2) to facilitate the de novo viral infection of SARS-CoV-2 while BRD4 is the least effective in this task [26,27,64]. Of note however, the BETi JQ1 and ABBV-744, when administered on the same day with SARA-CoV-2 infection in K1-hACE2 transgenic mice constitutively expressing the human ACE2 receptor, enhanced the viral replication [27]. These data underscore the antiviral role of BET proteins post entry and raise concerns about BET protein inhibition in ongoing SARS-CoV-2 infections.

Acute respiratory distress syndrome (ARDS) is a serious pathological condition that is associated with severe pulmonary inflammation, hypoxia, and edema [65]. It is also one of the major complications in severe COVID-19 patients [66]. The effects of BRD4 targeting in ARDS patients have been reported recently. In one study [28], BRD4 siRNA lipoplexes were found to suppress inflammatory infiltration of neutrophils and mast cells in the lungs of mice challenged with LPS. Blocking BRD4 also attenuated cytokine storm and oxidative stress associated with ARDS. Mechanistically, knockdown of BRD4 attenuated nuclear expression of NF-κB and STAT3, two critical downstream targets of TLR-4 signaling activated by LPS. The same group also reported the induction of BRD4 by LPS in the RAW264.7 macrophage cell line and the BEAS-2B bronchial epithelial cell line as well as in an LPS-promoted tumor metastatic model [29]. However, whether BET/BRD4 inhibition will block LPS-promoted tumor metastasis in vivo remains to be tested.

### 2.2. Asthma

The T helper 2 (Th2) cell-mediated inflammatory response in the airways represents a key mechanism underlying allergic asthma [67]. Although much is known about the pathogenesis of allergic asthma, the mechanisms of Th2 cell differentiation remain largely unclear. A very recent study unveiled a novel role of BRD4 in Th2 cell differentiation from mouse primary naïve CD4+ T cells [68]. BRD4 in collaboration with Polycomb repressive complex 2 (PRC2) repressed transcriptional expression of the Th2 negative regulators, Foxp3 and the E3-ubiquitin ligase Fbxw7. This in turn promoted lineage-specific differentiation of Th2 cells from mouse primary naïve CD4+ T cells. Specifically, Fox3p was found to repress the Th2-specific transcription factor Gata3, while Fbxw7 promoted ubiquitination-mediated protein degradation of Gata3. JQ1 treatment eliminated the repression of Foxp3 and Fbxw7 by the BRD4 BD2 domain. This effect leads to the activation of Gata3-regulated genes including IL-4, IL-5, and IL-13. This study provides evidence that BRD4 is involved in allergic asthma by modulating the Th2-mediated inflammatory response, which warrants further investigation.

IL-9-producing helper T cells (Th9) are characterized by IL-9 expression and are characteristic of allergic lung inflammation. A comprehensive study by Xiao and colleagues uncovered a novel role of BRD4 in Th9 cell induction and IL-9 production with implications for the treatment of airway inflammation with BRD4 inhibition [30]. The authors first showed that OX40 (CD134) robustly induced CD4+ T cell differentiation into Th9 cells accompanied by the assembly of super enhancers on the *Il9* locus using H3K27Ac as an indicator of active enhancers. They identified the enrichment of BRD4 to the *Il9* super enhancer region (H2K27Ac) in OX40-induced Th9 cells. Further, they found that JQ1 dramatically inhibited OX40 co-stimulation (with the Th9 cell medium containing TGF-β + IL-4)-induced Th9 cell conversion from naïve CD4+ T cells (reduced from 70% to 11% with JQ1). Consistent effects were found using siRNA targeting BRD4. Further, several mouse models were used to evaluate the effects of BRD4 inhibition on airway inflammation. These include the OX40Ltg spontaneous airway inflammatory mouse model, aerosolized OVA challenged mouse model (3 weeks after immunization with OVA in alum adjuvant), and an adoptive transfer model in Rag-1 deficient mice with the transfer of OT-II cells with selective BRD4 knockdown. BRD4 inhibition with JQ1 or BRD4 knockdown markedly reduced airway inflammation, as shown by the suppressed proliferation of mucin-producing cells in the airways and inflammatory cell infiltration into the lungs, as well as suppressed IL-9 levels in the bronchoalveolar lavage (BAL) compared to corresponding controls. These data together demonstrate the crucial role of BRD4 in mediating airway inflammation via IL-9 secretion and imply a novel strategy for targeting BRD4 in allergic airway inflammation.

The proinflammatory cytokine interleukin-17F (IL-17F) expression is also increased in the airways of asthmatics and correlates with asthma severity [69]. IL-17F induces the production of CXCL8 (IL-8) and the latter potentially contributes to neutrophilic infiltration into the airway. BRD4 has been reported to mediate CXCL8 production and CDK9 phosphorylation by IL-17F in human ASMCs [70], suggesting that BRD4 may be involved in neutrophilic infiltration by modulating IL-17F/CXCL8 signaling.

Airway smooth muscle cell (ASMC) hyperproliferation and secretion of proinflammatory mediators contributes to airway remodeling and inflammation in asthma [71,72,73]. The response of ASMCs from healthy subjects and asthmatics to BETi JQ1 and I-BET762 in the presence of fetal calf serum (FCS) and TGF-β1 stimulation has been reported previously [31]. The study found that the proliferation and secretion of IL-6 and CXCL8 of ASMCs from severe and non-severe asthmatics were more resistant to treatment with JQ1 and I-BET762. While c-Myc knockdown significantly inhibited the proliferation of ASMCs derived from both healthy and asthma donors, JQ1 treatment had no effect on c-Myc mRNA levels, a reported target of BRD4 [74,75]. Our previous work also supports c-Myc independent effects of JQ1 in the induction of cancer cell apoptosis [76], which seems to be cell-type dependent. Instead, JQ1 was found to increase p21 and p27 and potentially induce cell cycle arrest. In addition, JQ1 also attenuated the binding of BRD4 with the *CXCL8* promoter. CXCL8 is a chemokine that contributes to steroid-resistant neutrophilic airway inflammation [77]. Clifford and colleagues found that ASMCs from asthmatic patients secrete higher levels of CXCL8 compared to that from healthy donor controls [32]. They further found that ASMCs from asthmatic donors demonstrate increased histone H3 lysine acetylation (H3K18Ac) and increased binding of p300. BRD4 and BRD3 were found to bind to the *CXCL8* promoters, which can be inhibited by different BET inhibitors including PFI-1, I-BET, and JQ1. I-BET also disrupted the binding of BRD4 and RNA polymerase II to the *CXCL8* promoter, without affecting the binding of transcription factors, including NF-κB and C/EBPβ [78]. Conversely, in human bronchial epithelial cells, JQ1 impaired the binding of NF-κB p65 to the *CXCL8* promoter [49]. In viral infection, the vaccinia virus protein F14 was recently shown to selectively inhibit a subset of NF-κB signaling induced by TNF-α, i.e., suppressing BRD4 recruitment to the promoters of *CCL2* and *CXCL10*. However, the binding to the promoters of *NFKB1A* and *CXCL8* was not affected. Interestingly, JQ1 treatment blocked the induced binding of BRD4 to the promoters of *CCL2* and *CXCL10* but not to *NFKBIA* and *CXCL8* promoters [79]. These findings suggest bromodomain-independent recruitment of BRD4 to the promoters of certain genes, while the detailed mechanism(s) of this interaction remain(s) elusive.

The role of BRD4 in airway remodeling, another key feature of severe asthma, has gained much attention recently [80]. Two highly selective BRD4 inhibitors, ZL0420 and ZL0454, were compared to the non-selective pan-BET inhibitors, JQ1 and RVX208 (also called apabetalone), with respect to their ability to reduce chronic airway remodeling induced by TLR3 using a mouse model that mimics recurrent virus-induced asthma exacerbations [33]. The authors showed that BRD4 targeting by siRNA or specific BRD4 inhibitors (ZL0420 and ZL0454) dramatically suppressed TLR3-mediated mesenchymal transition in hSAECs, as indicated by the suppressed induction of genes including *SNAI1*, *ZEB1*, *IL6*, *VIM*, *FN1*, *MMP9*, and *COL1A* by poly(I:C) over 15 days. Both ZL0420 and ZL0454 inhibited the acetylation at histone lysine residue 122 (H3K122Ac) induced by Poly(I:C) in hSAECs. This is consistent with the reported intrinsic histone acetyltransferase (HAT) activity of BRD4 [81]. The HAT activity of BRD4 towards H3K122Ac can be attenuated by the BETi I-BET [81], suggesting the dependence on its binding to BDs. Further, the study examined the in vivo efficacy of BRD4 inhibitors on innate inflammation-driven airway remodeling. The authors found that BRD4 inhibitors blocked TLR3 agonist-induced epithelial to mesenchymal transition (EMT) in the lungs of mice receiving poly(I:C). Treated mice also demonstrated improved lung function and pathological changes, as well as reduced collagen deposition in subepithelial and interstitial spaces. This study provides evidence that BRD4 is a novel target for inflammation-induced airway remodeling. Whether BRD4 inhibition could reverse airway remodeling induced by pol(I:C) deserves future exploration and may inform the role of BRD4 targeting in inflammation-driven airway remodeling.

Recently, Lu and colleagues also reported the implication of BRD4 in fine particulate matter 2.5 (PM2.5)-induced airway hyperresponsiveness (AHR) [34]. In the nose-only PM2.5 exposure model, PM2.5 induced AHR, lung inflammation, and elevated expression of BRD4. Such effects were attenuated by the BRD4 inhibitor ZL0420. In vitro experiments showed that PM2.5 induced hASMC contraction and migration, as well as elevated expression of BRD4, vimentin, MMP2, and MMP9. Interestingly, these effects were reversed by ZL0420 and BRD4 siRNA in hASMCs. The data suggest that BRD4 contributes to PM2.5-induced AHR and may represent a therapeutic target for the treatment of inflammatory airway diseases.

Exacerbations of asthma are modulated by acute viral infection. Tian et al. [35] showed that RSV induces BRD4 to complex with NF-κB/RelA. By doing so, BRD4 modulates the assembly of CDK9 and RNA polymerase II formation on the promoters of IRF1, IRF7, and RIG-1, promoting their transcriptional elongation. In vivo, BRD4 inhibition with JQ1 blocked poly(I:C)- and RSV-induced neutrophilia, mucosal chemokine production and airway obstruction. In cat dander-induced asthma, Tian and colleagues [36] further showed that BRD4 was also induced to complex with NF-κB/RelA in primary human hSAECs. The binding activates BRD4’s atypical HAT activity, leading to inflammatory and profibrotic gene expression, which can be blocked by the BRD4 inhibitor ZL0454. ZL0454 also blocks epithelial mesenchymal transition, myofibroblast expansion, IgE sensitization, and fibrotic changes in airways of naïve mice associated with cat dander exposure. These findings together support the idea that BRD4 may serve as a potential target in exacerbated asthma, regardless of the causes.

In summary, different layers of evidence support a critical role of BRD4 in allergic asthma and airway remodeling. BRD4 affects multiple cell types to promote asthma by modulating the secretion of proinflammatory and profibrotic mediators, cell differentiation, EMT, and imbalance of proliferation/apoptosis of ASMCs. Emerging evidence strongly supports the targeting of BET/BRD4 in asthmatics in clinical trial testing.

### 2.3. Pulmonary Artery Hypertension (PAH)

PAH is a vascular disease that primarily affects the distal pulmonary arteries. It is characterized by increased inflammation, vasoconstriction, and hyperproliferation of smooth muscle cells with suppressed apoptosis within the arterial wall. It is a progressive disease that leads to pulmonary vascular resistance, right ventricle failure and death. Previously, Courboulin and colleagues compared 337 microRNAs (miRNAs) between PAH and control lungs and reported six upregulated miRNAs including miR21 and a single downregulated miRNA, namely miR-204 in PAH [82]. Later, Meloche and colleagues reported elevated BRD4 expression in the distal pulmonary arteries and pulmonary arterial smooth muscle cells (PASMCs) of PAH patients. Further, this enhanced expression was shown to be miR-204-dependent [37]. In addition, these investigators showed that JQ1 treatment reversed established PAH in the Sugen 5416/hypoxia rat model [37]. They found that BRD4 targeting by JQ1, or siRNA suppressed pro-survival signals (e.g., NFATc2, BCL-2 and survivin) and increased cell cycle arrest via p21 upregulation in PASMCs, which may account for the observed effects of JQ1 in vivo. The miR-204-BRD4 axis has also been noted in head and neck squamous cell carcinoma, in which miR-204 acts as a tumor suppressor by enhancing p27 mRNA stability through targeting BRD4 [83]. Another pan-BET inhibitor, I-BET151, was also shown to ameliorate right ventricle hypertrophy and pulmonary hypertension in rats induced by chronic hypoxia and pulmonary inflammation [38]. In addition, the BD2-selective BETi RVX208 also demonstrated benefits for PAH in the Sugen/hypoxia and monocrotaline (MCT)+shunt-PAH rat models [39]. RVX208 treatment reversed vascular remodeling and improved lung hemodynamics. RVX-208 also reduced the pressure load to the right vesicle in a rat PAH model induced by pulmonary artery banding. In vitro data showed that RVX-208 restored the altered phenotypes of proliferation, apoptosis resistance, and inflammation of both PASMCs and PAECs derived from PAH patients. The authors further identified two downstream targets of BRD4, FoxM1 and PLK1, implying the modulation of DNA damage response as a contributor to PAH [39]. The consistent finding was reported that JQ1 attenuated the inflammation (mRNA of IL-6 and CXCL8) and proliferation of human pulmonary microvascular endothelial cells (HPMECs) from healthy subjects. These observations are related to reduced NF-κB/p65 recruitment to the native *IL6* and *CXCL8* reporters and the induced cell cycle arrest at G0/G1 by JQ1 [40]. A recent pilot clinical trial in PAH patients also demonstrated the benefits of BET targeting. RVX-208 treatment for 16 weeks decreased pulmonary arterial resistance and increased cardiac output, stroke volume, and compliance in all six PAH patients [84]. Future larger and placebo-controlled trials are warranted to assess the efficacy of BETi in PAH patients. Together, these findings support further exploration of BRD4 as a novel therapeutic target in PAH.

### 2.4. Pulmonary Fibrosis

Largely due to the discovery of specific BET inhibitors [60,61], studies were enabled to explore the targeting of BET protein in pulmonary fibrosis. Two papers from the same research group emerged a few years later that investigated the role of BET proteins in pulmonary fibrosis, which represent a milestone for targeting BET/BRD4 in this context. In the first study, Tang and colleagues [41] showed that the response of human lung fibroblasts (HLFs) from healthy donors to TGF-β1 and PDGF-BB was mediated by BET proteins. Specifically, TGF-β1 induces the acetylation of lysine 5 of H4 (H4K5ac, a reported BRD4 binding site [85]), and the binding of BRD4 to the promoters of IL-6, α-smooth muscle actin (α-SMA), and plasminogen activator inhibitor-1 (PAI-1). Accordingly, pretreatment of HLFs with JQ1 and I-BET blocked TGF-β1-induced transcription of these genes. Further, TGF-β1- and PDGF-BB-mediated cell phenotype switching of HLFs, including proliferation, migration, and extracellular matrix production, was also abolished by JQ1 and I-BET pretreatment. Interestingly, knockdown of BRD2 and BRD4, but not BRD3, significantly blocked the induction of α-SMA by TGF-β1 in HLFs, indicating the differential effects of BET proteins in mediating TGF-β1-induced myofibroblast differentiation. Of note, JQ1 treatment did not affect Smad2/Smad3 nuclear translocation, suggesting a null effect on Smad3 phosphorylation. In the bleomycin model of pulmonary fibrosis, JQ1 treatment reduced total BAL fluid cells and collagen deposition, as indicated by hydroxyproline content in the lungs of injured mice. In the second paper [42], the authors compared the response of HLFs from idiopathic pulmonary fibrosis (IPF) lungs and tumor-free lungs (control) of tumor patients. They found that HLFs are more responsive or activated in response to PDGF-BB treatment, as determined by cellular proliferation, migration, and IL-6 release. However, JQ1 treatment inhibited these responses. Consistent with predictions from the in vitro evidence, JQ1 dose-dependently suppressed inflammatory infiltration in the lungs of bleomycin-challenged mice (21 days). In accord, pulmonary fibrosis, as indicated by collagen I expression, was also largely inhibited by JQ1. Recently, Bernau and colleagues [43] further showed the efficacy of selective inhibition of the BRD4 BD1 domain in reducing myofibroblast differentiation and reversing established pulmonary fibrosis in mice using the BRD4 BD1-selective inhibitor ZL0591.

CG223, a novel quinolinone-based BETi, was also reported to attenuate bleomycin-induced pulmonary fibrosis in mice [44]. When given daily in the inflammatory phase of the bleomycin model (3 to 12 days after bleomycin instillation), CG223 reduced the number of lymphocytes and neutrophils in BAL, collagen deposition in the lung, and the Ashcroft score of mice that received bleomycin. Specifically, bleomycin induced the enrichment of BRD4 in fibrotic lesions of the lungs of mice and the binding of BRD4 to the promoters of profibrotic genes including thrombospondin 1 (*Thbs1*), integrin β3 (*Itgb3*), and smooth muscle alpha (α)-2 actin (*Acta2*). In vitro experiments showed that CG223 dose-dependently inhibited TGF-β1-induced expression of these genes in primary lung fibroblasts isolated from untreated C56BL/6 mice. Because *Thbs1* and *Itgb3* genes are involved in the entry into the TGF-β1 autocrine/paracrine loop [86,87,88], this study implies the role of BRD4 in triggering this response. In addition to BRD4-mediated downstream gene expression of TGF-β1 as mentioned above, this study suggests another layer of interaction between BRD4 and TGF-β signaling.

Reactive oxygen species (ROS) are important mediators of TGF-β-induced myofibroblast differentiation. Inhibition of ROS production attenuates lung injury in bleomycin-challenged mice [89,90]. A gene array analysis [45] showed increased *NOX4* gene expression and decreased *SOD2* expression in systemic sclerosis (SSc) and IPF lung fibroblasts compared to non-fibrotic controls. In the same study, treatment with JQ1 was found to inhibit the TGF-β1-induced *NOX4* increase, *SOD2* decrease, and Nrf2 inactivation. In accord, the production of ROS and myofibroblast differentiation induced by TGF-β1 were also significantly blocked by JQ1 in HLFs. Further, the authors found that BRD3 and BRD4, but not BRD2, bind to the promoters of *NOX4* and mediate TGF-β1-induced NOX4 expression. In addition, the BRD4/NOX4 axis has been implicated in age-related pulmonary fibrosis [46]. Inhibition of BET with OTX015 resolves established pulmonary fibrosis in 18-month-old mice that received bleomycin for 21 days. Lung function and histology were largely restored by OTX015 treatment for 21 days after the bleomycin challenge. In a brief report [47], JQ1 treatment was found to upregulate genes enriched in glutathione metabolism and downregulate fibrosis-related genes in primary HLFs from an IPF patient via an RNA-sequencing analysis. These studies suggest that BET proteins are also involved in regulation of oxidative/reductive balance to promote myofibroblast differentiation.

Besides IPF, radiation-induced pulmonary fibrosis (RIPF) may likewise involve BET protein regulation. RIPF has been reported to occur in about 16% of Hodgkin’s lymphoma patients [91] and in 70–80% of lung cancer patients who received high-dose radiation [92]. In a study evaluating the effect of JQ1 in RIPF [48], the authors found that JQ1 protected normal lung tissue after irradiation, attenuated RIPF, lung inflammation, and collagen deposition in a rat model of pulmonary damage with 20Gy radiation. Proteins, including BRD4 and c-Myc in the lungs of irradiated mice, were reduced by JQ1, as well as collagen, TGF-β1, p-NF-κB p65, and p-Smad2/3. JQ1 also repressed the radiation-induced myofibroblast differentiation of normal HLFs. These results provide evidence supporting the targeting of BET/BRD4 as a candidate-effective strategy for the treatment of different forms of pulmonary fibrosis.

### 2.5. Chronic Obstructive Pulmonary Disease (COPD)

COPD is the third leading cause of death worldwide and appears to have an increasing trajectory [93]. Currently, there is no approved drug that can cure COPD. The pathogenesis of COPD is characterized by chronic inflammation and oxidative stress. A previous study [49] showed that the BET inhibitors JQ1 and PFI-1 dramatically reduced IL-6 and CXCL8 expression in human epithelial cells stimulated by IL-1β plus H_2_O_2_. JQ1 also inhibited the recruitment of p65 and BRD4 to the promoters of *IL-6* and *CXCL8*. BRD4, but not BRD2, was found to mediate IL-6 and CXCL8 release from human primary epithelial cells. The results suggest a role for BRD4 in inflammation- and oxidative-stress-related proinflammatory cytokine production. Consistently, JQ1 treatment was also reported to inhibit pro-inflammatory gene expression in alveolar macrophages from COPD patients [50]. Alveolar macrophages extracted from COPD transplanted lungs were stimulated with LPS in the presence or absence of JQ1. While LPS induced a proinflammatory M1 macrophage phenotype, JQ1 treatment reversed these changes. There was no difference between the expression of BET proteins (BRD2, 3, 4, and BRDT) in alveolar macrophages from COPD patients and control subjects. In another study [51], JQ1 treatment ameliorated the oxidative stress of primary ASMCs, monocytic cells and the THP-1 cell line. JQ1 activates the Nrf2-dependent transcription and expression of antioxidants including hemeoxygenase-1 (HO-1), NADPH quinone oxidoreductase 1 (NQO1), and the glutamate-cysteine ligase catalytic subunit (GCLC). It also blocked H_2_O_2_-induced intracellular ROS production. All BET proteins (BRD2, 3, and 4) were found to interact with the Nrf2 protein. BRD2 and BRD4 were also found to bind to the promoters of *NQO1* and *HO-1* genes. Interestingly, neither the interaction between BET proteins and Nrf2 nor the binding of BET proteins to the promoters of antioxidant genes (*NQO1* and *HO-1*) was affected by JQ1 treatment, suggesting BD-independent activity of these BET proteins. These results indicate that BET proteins can selectively modulate Nrf2-specific gene expression and thus may contribute to oxidative-stress-related diseases such as COPD.

Small airway fibrosis occurs in COPD patients and contributes to obstructed air flow. The response of ASMCs derived from COPD patients to a profibrotic stimulus, e.g., TGF-β1, differs from those in healthy controls. Work by Zakarya and colleagues showed that there was higher mRNA expression of *COL15A1* and *TNC* (tenascin C) in ASMCs from COPD patients compared to non-COPD controls [52]. Accordingly, elevated collagen 15α1 and TNC staining were found in the lungs of COPD small airways in contrast to those from non-COPD controls. TGF-β1 treatment induced a more pronounced increase in *COL15A1* and *TNC* mRNA levels in ASMCs from COPD smokers versus non-COPD smokers, which were blocked by JQ1 treatment. JQ1 treatment was also found to abolish H4 acetylation at the promoters of *COL15A1* and *TNC* induced by TGF-β1 only in COPD ASMCs. By contrast, H3 acetylation at the promoters of these two genes was minimally induced by TGF-β1, suggesting differential regulation. Findings from this study provide a novel insight into the implication of BET proteins in TGF-β1-induced specific gene expression and histone H4 acetylation. Whether these effects are specific to BRD4 remains to be tested since only the pan-BET inhibitor JQ1 was used. Again, these findings in human ASMCs warrant further validation in the in vivo setting of COPD.

In a cigarette-smoke- and LPS-induced COPD model in mice [53], JQ1 treatment reversed histopathological changes and the induced cytokine profile. The mean linear intercept, destructive index, and inflammatory score of mice with induced COPD were dose-dependently improved by JQ1. JQ1 treatment also reduced the expression of MMP2, MMP9, IL-1β, IL-17, IL-6, and TNF-α that were enhanced in COPD mice. In addition, the oxidative stress in COPD mice as indicated by increased MDA levels and decreased SOD, HO-1, and T-AOC, was also significantly improved by JQ1. These observed effects of JQ1 are associated with suppressed nuclear NF-κB p65, p65 acetylation (Lys310), and p65/DNA binding activity. This study demonstrates the efficacy of BETi in reversing established preclinical COPD. During viral exacerbation, researchers recently noticed elevated mRNA expression of BRD4 in the blood and sputum of COPD patients compared to those in a stable state [54]. Consistently, elevated BRD4 expression was found in the lungs of mice undergoing influenza infection and cigarette smoke exposure. This model also demonstrated inflammatory cells infiltration, IL-6, and chemokines induction in the lungs. Concurrent JQ1 treatment dramatically suppressed these alterations in the mice. Further, the authors showed that BRD4 siRNA significantly inhibited the protein and mRNA levels of IL-6 and CXCL8 induced by cigarette smoke exposure and influenza virus infection in the bronchial epithelial cell line, BEAS-2B. These two studies support the concept that BRD4 may be a target in cigarette smoke- and infection-exacerbated COPD.

**Figure 1:**
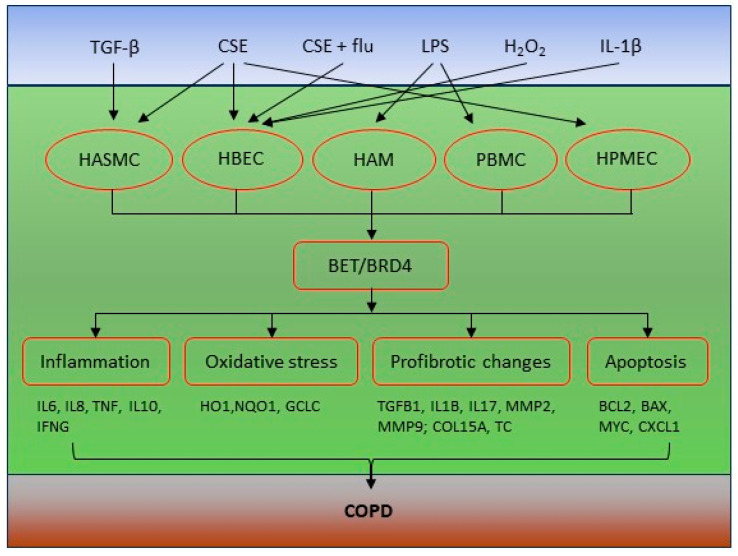
Schematic diagram of BET/BRD4 in the pathogenesis of COPD. A variety of stimuli are involved in the regulation of multiple cell types that are implicated in the pathogenesis of COPD. BET protein, particularly BRD4, plays important roles in these cell types to regulate key cellular processes including inflammation, oxidative stress, profibrotic changes, and apoptosis. These deregulated processes and related genes together contribute to the development of COPD. CSE, cigarette smoke extract; CXCL1, C-X-C motif chemokine ligand 1; GCLC, glutamate-cysteine ligase catalytic subunit; HAM, human alveolar macrophage; HASMC, Human airway smooth muscle cell; HBEC, human bronchial epithelial cell; HO1, heme oxygenase-1; HPMEC, human pulmonary microvascular endothelial cell; NQO1, NAD(P)H quinone dehydrogenase 1; and TNC, tenascin C.

MiRNAs are known to be involved in the regulation of inflammation in different contexts. In COPD, several non-coding RNAs including circulating RNA (circRNA), long non-coding RNA (lncRNA), and miRNA have been reported to regulate airway epithelial cell apoptosis and inflammation induced by cigarette smoke. For example, both the circRNA ankyrin repeat domain 1 (circANKRD11) [55] and circRNA oxysterol binding protein like 2 (circOSBPL2) [56] were found to increase in the lungs of smokers with or without COPD. Downregulation of either mitigates cigarette smoke-induced apoptosis, inflammation, and oxidative stress in human bronchial epithelial cells. Mechanistic links to BRD4 were found for both circANDRD11 and circOSBPL2, with miR-I45-5p and miR-193a-5p as the intermediate targets, respectively. In addition, miR-218 [57] and miR-29b [58] are also associated with cigarette smoke extract (CSE)-induced apoptosis and inflammation in human bronchial epithelial cell lines. Both miRNAs were downregulated in COPD lungs, which correlate with lung function and inflammation. In contrast, BRD4 was found to be increased in COPD patients and induced by CSE. Knockdown of BRD4 with siRNA blunted the CSE-induced inflammatory cytokine expression and secretion (IL-6, CXCL8, and TNF-α). In addition, the long noncoding MIR155 host gene (MIR155HG) was found to inversely correlate with miR-218-5p in COPD lungs and CSE-induced HPMECs [59]. Further, LncRNA MIR155HG was found to downregulate miR-128-5p expression, through which BRD4 was targeted indirectly. In conclusion, the LncRNA MIR15HG/miR-128-5p/BRD4 axis was implicated in the cell apoptosis and inflammation of HPMECs. Despite these findings, the regulation of BRD4 by non-coding RNAs in the pathogenesis of COPD remains largely unclear and an open research avenue. A schematic view of BRD4 in the pathogenesis of COPD is shown in Figure 1. BRD4 contributes to the development of COPD by regulating multiple cellular processes in various cell types. Targeting BRD4 may represent a novel, effective strategy for the treatment of COPD.

## 3. Conclusions and Future Perspectives

In summary, BRD4 is involved in the development of several major lung diseases. This is related to its role as a scaffold protein to enhance transcriptional activity. However, the molecular mechanisms underlying BET/BRD4 in different lung diseases remain largely unclear. For instance, the gene specificity of BRD4 binding, the coordinated or redundant role of BET proteins, and the preferred downstream targets of BET/BRD4 in different lung diseases remain unclear. In addition to the reported binding proteins (e.g., the Mediator [94] and pTEFb complex [1]), identification of novel BRD4 partners, for example through interactome analysis, may uncover novel functions of BRD4 in disease progression. Beyond the role of an epigenetic reader, BRD4 may also regulate gene transcription through its atypical histone acetylation activity and kinase activity [95], adding another aspect of the control of gene transcription. Further, BRD4 integrates in the pathogenesis of lung diseases also through manipulating innate immunity and adaptive immune response. Future single cell RNA sequencing analysis may help identify subgroups of patients that benefit from BET/BRD4 targeting in different settings. While BETi has been actively tested in human diseases including cancer and metabolic diseases, clinical trials for testing BET/BRD4 inhibitors in lung diseases are supported by currently available evidence and could potentially identify safe, new and more effective interventions.

## Figures and Tables

**Table 1:** BET proteins in pulmonary diseases.

BET Protein	BET Inhibition	Cell Type	Treatment	Target Gene	Signal Pathway	In Vivo Model	Ref.
Acute lung inflammation							
BRD4	ZL0454	A549, human small airway epithelial cell	RSV	JUN, FOSL1	AP-1	N/A	[11]
N/A	CPI-203	Human bronchial epithelial cell	N/A	Th17 cytokines (e.g., IL17A, IL22)	N/A	*Pseudomonas aeruginosa* infection mouse model	[21]
BRD2, BRD4	JQ1, shRNA	Human umbilical vein endothelial cell; leukocyte	TNF-α	IL6, IL8	NF-κB, p38, JNK MAPK	LPS mouse model	[22]
N/A	IBET762, JQ1, RVX-297	Human U937, PBMC, and fibroblast; mouse B cell and BMDM	LPS	IL6, IL17A	N/A	Rat and mouse collagen-induced arthritis model; mouse collagen antibody-induced arthritis model; LPS mouse model	[23]
BRD4	ZL0420, ZL0454, JQ1, RVX-208	Human small airway epithelial cell	Poly(I:C)	ISG54, ISG56, IL8, CXCL2	N/A	Poly(I:C) mouse model	[24]
BRD2, BRD4	N/A	HEK-293T/17	SARS-CoV-2	N/A	N/A	N/A	[25]
N/A	JQ1, OTX015, ZBC260	Murine lung bronchial and nonbronchial cells, LNCaP, H1437	SARS-CoV-2	ACE2, TMPRSS2	N/A	N/A	[26]
BRD2, BRD3, BRD4	JQ1, dBET6, ABBV-744	HEK293T, A549, Calu3	SARS-CoV-2	IFNB1, ISG15, IL6	N/A	K18-hACE2 mouse model	[27]
BRD4 upregulation	BRD4 siRNA	RAW 264.7 and BEAS-2B	LPS	IL-1β, IL6, IL17A, IL22	NF-κB, STAT3, Akt/mTOR/MAPK	LPS-induced mouse model; xenograft mouse model	[28,29]
Asthma							
BRD4	JQ1, BRD4 siRNA	Th9 cell	OX40	IL9	NF-κB	Acute allergic lung inflammation mouse model; adopt transfer mouse model	[30]
BRD4	JQ1, I-BET762, BRD4 siRNA	Human airway smooth muscle cell	FCS + TGF-β	IL6, IL8	N/A	N/A	[31]
BRD3, BRD4	JQ1, PFI-1, I-BET	Human airway smooth muscle cell	N/A	IL8	RNA Pol II binding	N/A	[32]
BRD4	ZL0420, ZL0454, JQ1, RVX208	Human small airway epithelial cell; human lung fibroblast	Poly(I:C)	N/A	N/A	Poly(I:C) mouse model	[33]
BRD4 upregulation	ZL0420, BRD4 siRNA	Human airway smooth muscle cell	PM2.5	MMP2, MMP9	N/A	PM2.5 challenged mouse model	[34]
BRD4	JQ1, BRD4 siRNA	Human small airway epithelial cell	RSV	IRF1, IRF7, RIGI, Il6, Cxcl1, Cxcl2	NF-κB	Poly(I:C) mouse model; RSV infection mouse model	[35]
BRD4	ZL0454	Human small airway epithelial cell	Cat dander	SNAI1, CDH1, Acta2, Fn1, Cxcl1, Il6, Vim, Col1a1	NF-κB	Cat dander exposed mouse model	[36]
PAH							
BRD4 upregulation	JQ1, BRD4 siRNA	Pulmonary artery smooth muscle cells	N/A	CDKN1A, NFAT, BCL2, BIRC5	N/A	Sugen/hypoxia rat model	[37]
N/A	I-BET151	N/A	N/A	N/A	N/A	LPS plus hypoxia rat model	[38]
BRD4 upregulation	RVX-208, BRD4 siRNA	Human pulmonary microvascular endothelial cell, human pulmonary microvascular smooth muscle cell	TNF-α	FoxM1, PLK1	N/A	Sugen5416 + hypoxia rat PAH model; monocrotaline + shunt PAH model; Pulmonary artery banding rat model	[39]
N/A	JQ1	Human pulmonary microvascular endothelial cell	FCS	IL6, IL8, CDKN1A	NF-κB	N/A	[40]
Pulmonary fibrosis							
BRD4	JQ1, I-BET	Human lung fibroblast	TGF-β, PDGF-BB	ACTA2, IL6, PAI1	N/A	Bleomycin challenged mouse model	[41]
BRD4	JQ1	Human lung fibroblast	TGF-β, PDGF-BB	IL6, IL8, CDKN1A	N/A	Bleomycin challenged mouse model	[42]
BRD4	ZL0591	N/A	N/A	N/A	N/A	Bleomycin challenged mouse model	[43]
BRD4 upregulation	CG223	mouse lung fibroblast	TGF-β	Thbs1, Itgb3, Acta2	N/A	Bleomycin challenged mouse model	[44]
BRD2, BRD3, BRD4	JQ1	Human lung fibroblast	TGF-β	NOX4, SOD2	N/A	N/A	[45]
BRD4	JQ1, I-BET762, OTX015	Human lung fibroblast	TGF-β	NOX4	N/A	Aged mice challenged with bleomycin	[46]
N/A	JQ1	Human lung fibroblast and myofibroblast	TGF-β	ACTA1, FN1	N/A	N/A	[47]
BRD4 upregulation	JQ1	Human lung fibroblast	Radiation	MYC, TGFB1	Smad2/3, NF-κB	Radiation-induced pulmonary fibrosis	[48]
COPD							
BRD2, BRD4	JQ1, PFI-1	Human bronchial epithelial cell	H_2_O_2_, IL-1β	IL6, IL8	NF-κB	N/A	[49]
N/A	JQ1	Human alveolar macrophages, peripheral blood mononuclear cells (PBMC)	LPS	Cell- and time-dependent genes, not listed here	N/A	N/A	[50]
BRD2, BRD3, BRD4	JQ1	Human airway smooth muscle cell, THP-1 cell, PMBC	CSE	HO1, NQO1, GCLC	Nrf2	N/A	[51]
N/A	JQ1	Human airway smooth muscle cell	TGF-β	COL15A1, TNC	N/A	N/A	[52]
N/A	JQ1	N/A	N/A	Mmp2, Mmp9, Ifng, Il17, Il1b, Il6, Tnf, Il10	NF-κB	CSE plus LPS induced mouse model	[53]
BRD4 upregulation	JQ1	BEAS-2B	CSE plus flu	IL6, IL8, CXCL1	N/A	CSE plus flu infection mouse model	[54]
BRD4 upregulation	N/A	Human pulmonary microvascular endothelial cell	CSE	BCL2, BAX, TNF, IL1B, IL6	CircANKRD11/miR-145-5p/BRD4	N/A	[55]
BRD4 upregulation	N/A	Human bronchial epithelial cell (16HBE)	CSE	IL8, IL1B, TNF	Circ-OSBPL2/miR-193a-5p/BRD4	N/A	[56]
BRD4 upregulation	BRD4 siRNA	BEAS-2B	CSE	IL8, IL1B, TNF	miR-218/BRD4	N/A	[57]
BRD4 upregulation	BRD4 siRNA	Human bronchial epithelial cell (HBE4-E6/E7)	CSE	IL6, IL8	miR-29b/BRD4	N/A	[58]
BRD4	N/A	Human pulmonary microvascular endothelial cell	CSE	BCL2, BAX, IL6, IL8, TNF	MIR155HG/miR-218-5p/BRD4	N/A	[59]

Note: N/A, not assessed.

## Data Availability

Not applicable.
